# A novel truncated fusion gene *ETV6*::AC010198.2 in post-MPN acute myeloid leukemia caused by del(12)(p13p11)

**DOI:** 10.3389/fonc.2025.1495182

**Published:** 2025-02-11

**Authors:** Jiao Lu, Guanqun Yang, Qiurong Zhang, Man Wang, Lijun Wang, Lu Chen, Haoyue Chen, Xin Jiang, Chunhua Liu, Tong Lin, Qiaoyan Han, Zefa Liu, Shi Zhen, Zhao Zeng, Jinlan Pan, Jiannong Cen, Suning Chen, Zheng Wang, Xingxia Zhang, Miao Sun

**Affiliations:** ^1^ Department of Hematology, Jingjiang People’s Hospital Affiliated to Yangzhou University, Taizhou, Jiangsu, China; ^2^ National Clinical Research Center for Hematologic Diseases, Jiangsu Institute of Hematology, the First Affiliated Hospital of Soochow University, Soochow University, Suzhou, China; ^3^ Changshu Hospital Affiliated to Soochow University, Changshu No.1 People’s Hospital, Suzhou, Jiangsu, China; ^4^ Department of Hematology, The Affiliated Zhangjiagang Hospital of Soochow University, Suzhou, Jiangsu, China; ^5^ Institute of Blood and Marrow Transplantation, Collaborative Innovation Center of Hematology, Soochow University, Suzhou, China; ^6^ Department of Hematology, People’s Hospital of Xinghua City, Taizhou, Jiangsu, China; ^7^ Department of Hematology, Suzhou Jsuniwell Medical Laboratory, Suzhou, China; ^8^ Department of Hematology, Huai’an Hospital Affiliated to Xuzhou Medical College and Huai’an Second People’s Hospital, Huai’an, Jiangsu, China

**Keywords:** AC010198.2, novel, MPN, AML, *ETV6*

## Abstract

The gene ETV6 has been confirmed to be a genetic susceptibility gene for thrombocytopenia and leukemia. Here, we report a long-chain noncoding RNA AC010198.2 as a novel fusion partner of ETV6, showing a karyotype of del(12)(p13p11), with poor prognosis in a post-MPN AML that has never been reported, which may be an vital initial event in the transformation of MPN to AML and deterioration of disease.

## Introduction

The *ETV6* gene, located at chromosome 12p13, belongs to the ETS family of transcription factors, which share a conserved 80 amino acid DNA-binding domain, the ETS domain (1). It encodes an essential transcriptional repressor that is abundantly expressed in hematopoietic stem and progenitor cells (HSPCs). It is critical for the maintenance of HSPCs and the formation of platelets (2, 3), and has been shown to be closely associated with inherited thrombocytopenia and leukemia predisposition (3, 4). *ETV6* variants include insertion/deletion mutations and structural variants (deletions, rearrangements) that may lead to truncation and loss of function of *ETV6*, ultimately contributing to thrombocytopenia and leukemia (4). It has been reported that *ETV6* has over 30 partner genes (**Supplementary Table 1**), such as *PDGFRB* (1), *RUNX1* (5), *ACSL6* (6), and non-protein coding RNA such as LINC02260 (7), and is one of the most common translocation genes in acute lymphoblastic leukemia (ALL) and other hematological disorders such as acute myeloid leukemia (AML) and myelodysplastic syndrome (MDS) (1). Functional investigations of *ETV6* variants have shown that they are associated with disrupted expression of genes involved in platelet production and platelet cytoskeleton dynamics, including the *CDC42* and *RHOA218* genes, but the mechanisms driving leukemia remain unclear (4). Recent studies have shown that *ETV6* is required to repress inflammatory gene expression in HSPCs and this mechanism may be critical for maintaining HSPC function (8). Here, we report a long-chain non-coding RNA AC010198.2 as a novel fusion partner of *ETV6*, showing a karyotype of del(12)(p13p11), with poor prognosis in a post-myeloproliferative neoplasm Myeloproliferative Neoplasms (MPN) AML that has never been reported.

A 61-year-old male patient was diagnosed with myelofibrosis in 2018 and treated with hydroxyurea in a local hospital. In June 2020, he was admitted to the First Affiliated Hospital of Soochow University for a bone marrow (BM) examination which further confirmed the diagnosis of myelofibrosis with CALR exon9 gene mutation and karyotype of 48, XY, +Y, +21[10]. He was instructed to take ruxolitinib 20mg twice a day for treatment and was then lost to follow-up.

**Figure 1:**
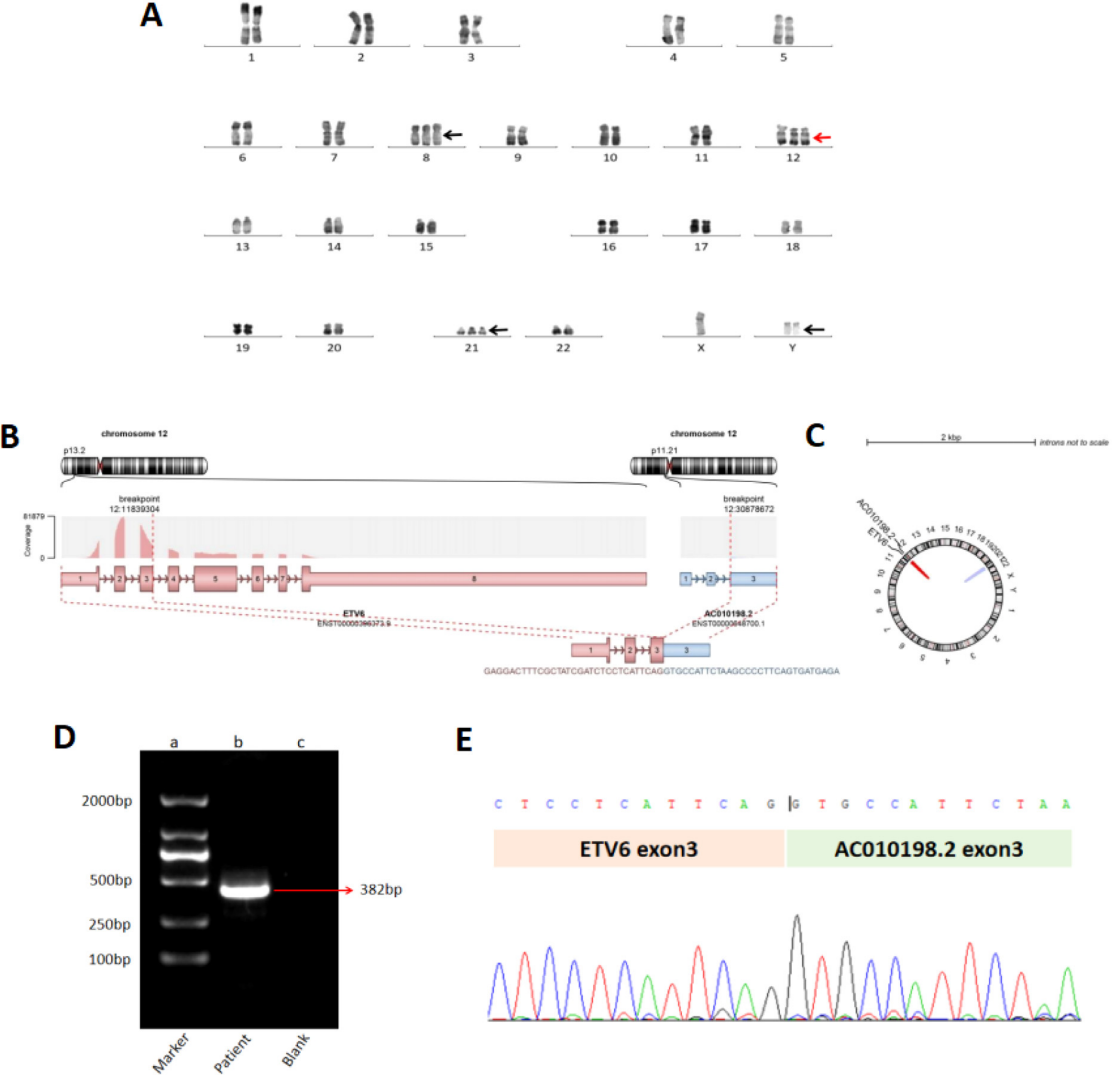
Laboratory examinations during the progression of the disease. **(A)** R-band karyotyping shows 47, XY, +21[1]/48, idem, +Y, del(12)(p13p11)[3]/49, idem, +Y, add(14)(p15), del(12)(p13p11), +del(12)(p13p11)[5]/50, idem, +Y, +8, del(12)(p13p11), and +del(12)(p13p11)[1]. **(B)** Schematic of the ETV6::AC010198.2 fusion breakpoint identified in this case. **(C)** A Circos plot. **(D)** RT-PCR for the ETV6-AC010198.2 fusion transcript with ETV6-F and AC010198.2-R primers and the expected product size was 382 bp. Lane a: 2000bp DNA ladder marker. Lane B: patient sample. Lane C: blank control. **(E)** Sanger sequencing of the PCR product confirmed the fusion between exon3 of the ETV6 gene and exon3 of the AC010198.2 gene.

On 29 March 2022, the patient was referred to a local hospital for routine peripheral blood (PB) tests indicating a white blood cell count of 111.7×10^9/L with neutrophils 45.70×10^9/L, hemoglobin 8.6 g/dL, and thrombocytes 167×10^9/L. PB smear showed 33% of primitive monocytes and 19% myeloblasts. BM aspiration revealed 20% primitive monocytes with intracytoplasmic Auer bodies. Flow cytometry for BM cells indicated 27.55% of abnormal myeloblasts. Conventional R-band karyotype analysis suggested a karyotype of 48, XY, +Y, del(12)(p12), +21[20]. The BCR-ABL fusion gene and MPN-related gene (CALR, JAK2, MPL) mutations were negative. He was diagnosed with post-MPN AML and treated with HA (harringtonine and cytarabine) in combination with hydroxyurea and ruxolitinib. The first course of treatment failed to achieve remission, and next-generation sequencing (NGS) showed *SRSF2*, *SETBP1*, *RUNX1*, and *IDH2* gene mutations which are reported to be enriched in post-MPN AML (9). Complete remission was still not achieved after the second D-HA (decitabine, homoharringtonine, and cytarabine) regimen immediately after the first course of treatment, and the gene mutations were persistently positive with a 1.23% relative expression level of the *WT1* gene and a karyotype of 46, XY, del(7)(q22q35)[4]/46, XY[6]. A third course of azacitidine in combination with a venetoclax regimen resulted in a minimal residual disease (MRD) of 2.1×10^-4^ but there was a rapid relapse in a short time later with more complex chromosomal abnormalities of 49, XY, +Y, add(4)(p15), del(12)(p13p11), +del(12)(p13p11),?der(17)t(17);?(p11);?, +21[8]/46, XY[2] found on 12 October 2022. Soon thereafter the karyotype got even more complicated and was found to be 47, XY, +21[1]/48, idem, +Y, del(12)(p13p11)[3]/49, idem, +Y, add(14)(p15), del(12)(p13p11), +del(12)(p13p11)[5]/50, idem, +Y, +8, del(12)(p13p11), +del(12)(p13p11)[1]. This was detected on 7 November 2022. Furthermore, the relative expression level of *WT1* increased to 44.08%. Targeted RNA sequencing showed a novel fusion composed of exons 1 to 3 of *ETV6* and exon 3 of the AC010198.2 (**Figure 1**) that mapped to chromosome 12p13.2 and chromosome 12p11.21, respectively. AC010198.2 is a long-chain non-coding RNA, a type of lncRNA, which contains three non-coding exons and does not encode any proteins. The ETV6-AC010198.2 transcript is not expected to produce a chimeric protein but results in a truncated or possibly unproductive ETV6 protein that contributes to the inactivation of the *ETV6* gene and thus promotes disease progression.

Chromosomal karyotype is a highly significant predictor of leukemic progression in MPNs. A number of chromosomal abnormalities, including i(17q), inv(3)/3q21, monosomy 7, 12p-/12p11.2, 11q-/11q23, and autosomal trisomy (excluding +8/+9), have been reported to be associated with a poorer prognosis and a nearly two-fold increased risk of leukemic transformation compared to other traditionally unfavorable karyotypes. The median overall survival for patients with these abnormalities is only 1.2 years (9, 10). This patient had autosomal trisomy +21 at the MPN stage and transformed into AML upon the appearance of 12p-, followed by progressively complicated karyotypes (**Table 1**), which resulted in rapid disease deterioration and poor treatment response. It is notable that 12p- was consistently positive during disease progression, with the exception of transient negativity observed following the initial treatment. This suggests that 12p- and the resulting fusion ETV6::AC010198.2 is an important factor contributing to genome instability and plays a pivotal role in the leukemic progression.

**Table 1:** Karyotypes at different stages of disease progression.

Detection date	Diagnosis	Disease state	Karyotype
2020/6/3	MF	Before AML	48,XY,+Y,+21[10]
2022/3/29	Post-MPN AML	At diagnosis of AML	48,XY,+Y,del(12)(p12),+21[20]
2022/5/20		After chemotherapy, non-remission	46,XY,del(7)(q22q35)[4]/46,XY[6].
2022/10/12		MRD: 13.2%	49,XY,+Y,add(4)(p15),del(12)(p13p11),+del(12)(p13p11),?der(17)t(17);?(p11);?,+21[8]/46,XY[2].
2022/11/7		MRD: 33.29%	47,XY,+21[1]/48,idem,+Y,del(12)(p13p11)[3]/49,idem,+Y,add(14)(p15),del(12)(p13p11),+del(12)(p13p11)[5]/50,idem,+Y,+8,del(12)(p13p11),+del(12)(p13p11)[1].

MF, myelofibrosis; MPN; AML, acute myeloid leukemia; MRD, minimal residual disease.

A thorough review of the extant literature reveals a paucity of studies addressing the AC010198.2 lncRNA. A single study found that high expression of AC010198.2 was associated with the sensitivity of cervical cancer cells to cisplatin (11). However, ongoing research continues to investigate the pathogenic mechanisms of the ETV6 gene in hematological disorders. In view of the considerable number of ETV6 partner genes, some researchers have broadly classified these fusion genes into three groups: 1) protein tyrosine kinases, 2) transcription factors, and 3) “unproductive” fusions, i.e., fusion that do not seem to produce a meaningful fusion protein (1). The most prevalent fusion, ETV6::RUNX1, has been shown to promote ALL by impeding hematopoietic reconstitution and lymphocyte differentiation through the expression of fusion proteins (5). The ETV6::ACSL6 fusion gene, which occurs almost exclusively in AML, is not expected to produce fusion proteins. However, it has been demonstrated to trigger an increase in the transcription of the encoded inflammatory factors (e.g., IL-3) adjacent to and distal to the breakpoint through the translocation and activation of a super-enhancer located at the *ETV6* gene, which ultimately leads to eosinophilia (12). The novel fusion gene ETV6::AC010198.2 reported in this study is similar to ETV6::ACSL6 in that it fails to produce fusion proteins. It is hypothesized that they may share analogous pathogenic mechanisms that stimulate the expression of neighboring genes under the super-enhancer of ETV6. Since this is the first international report, its pathogenic mechanism needs to be further verified.

In summary, we identified a novel ETV6 fusion gene transcript that does not express a functional protein in a post-MPN AML patient with del(12)(12p13p11) which may be a vital initial event in the transformation of MPN to AML and deterioration of disease.

## Data Availability

The datasets presented in this study can be found in online repositories. The names of the repository/repositories and accession number(s) can be found in the article/**Supplementary Material**.
