# Supplementary material for: A novel truncated fusion gene ETV6::AC010198.2 in post-MPN acute myeloid leukemia caused by del(12)(p13p11)
Source: Front Oncol. 2025 Feb 11;15:1495182. doi: 10.3389/fonc.2025.1495182 (PMC11850314; doi:10.3389/fonc.2025.1495182)
Supplement: Supplementary file 1 [file Table1.docx]

| **Supplementary Table 1: Partner genes known to fusion with ETV6** | | | | | | |
| --- | --- | --- | --- | --- | --- | --- |
| Fusion partner | Translocation | Disease | Relavant fusion protein | Mechanisms in cancer | Frequency | Reference |
| RUNX1 | t(12;21)(p13;q22) | ALL | ETV6/RUNX1 | ETV6/RUNX1 fusion protein impaires the hematopoietic reconstitution and lymphoid differentiation potentials. | 25% of ALL | (1) |
| PAX5 | t(9;12)(q11;p13) | B-ALL | PAX5/ETV6 | PAX5/ETV6 fusion protein was implicated in preB-cell receptor signaling and migration/adhesion. | 1% of ALL | (2-4) |
| STL | t(6;12)(p13;q23) | B-ALL | ETV6/STL? | STL overexpression? | One case | (5) |
| TTL | t(12;13)(p13;q14) | ALL | ETV6/TTL, TTL/ETV6 | Overexpression of neighboring gene? | One case | (6) |
| FGFR3 | t(4;12)(p16;p13) | Peripheral T-cell lymphoma secondary AML | ETV6/FGFR3 | Phosphatidylinositol 3-kinase (PI3-K) signal pathway | One case | (7, 8) |
| JAK2 | t(9;12)(p24;p13) | Pre-B ALL; Ph-like B-ALL; T-ALL; AML; aCML; MDS; MPN | ETV6/JAK2? | Unknown | 12 cases | (9) |
| ABL1 | t(9;12)(q34;p13) | ALL; AML; MPAL; MPN | ETV6/ABL1 | Unknown | 126 cases ( 65 MPN; 48 ALL; 12 AML; 1 MPAL) | (10) |
| ABL2 | t(1;12)(q25;p13) | T-ALL; AML-M3,-M4 | ETV6/ABL2 | Unknown | Three cases | (11-13) |
| NTRK3 | t(12;15)(p13;q25) | Congenital fibrosarcoma; mesoblastic nephroma; secretory breast carcinoma; salivary gland carcinomas; ALL; AML | ETV6/NTRK3 | Unknown | 0.71% of solid tumour cancer; 1% Ph-like ALL | (14, 15) |
| ARNT | t(1;12)(q21;p13) | T-ALL; AML-M2; MDS | ETV6/ARNT | Unknown | Three cases | (16-18) |
| MN1 | t(12;22)(p13;q11) | AML; MDS | MN1/ETV6? | MN1 overexpression | 17 cases | (19, 20) |
| BTL | t(4;12)(q11-q12;p13) | AML-M0 | ETV6/BTL or no fusion protein | Ectopic expression of the homeobox gene GSH2 | Five cases | (21, 22) |
| CDX2 | t(12;13)(p13;q12) | AML | ETV6/CDX2 or no fusion protein | Ectopic expression of the homeobox gene CDX2 | One case | (23) |
| HLXB9 | t(7;12)(q36;p13) | Infant AML | HLXB9/ETV6 | HLXB9 overexpression | 1.4% of infant AML | (24, 25) |
| ACSL6/ACS2 | t(5;12)(q31;p13) | AML; MDS; AEL | None | Super-Enhancer Triggers Inflammatory Factor (e.g., IL-3) Transcription | 17 cases | (26-28) |
| PER1 anti-sense | t(12;17)(p13;p13) | AML | ? | Overexpression of neighboring gene? | One case | (29) |
| PDGFRA | t(4;12)(q12;p13) | AML | ? | Unknown | Five cases | (30) |
| SCFD2 | t(4;12)(q12;p13) | AML | ? | Unknown | Two cases | (30) |
| GSX2 | t(4;12)(q12;p13) | AML | ? | Unknown | Three cases | (30) |
| PDGFRB | t(5;12)(q31;p13) | CMML;  T-lymphoblastic lymphoma; Chronic eosinophilic leukemia | ? | Unknown | Three cases | (31-33) |
| SYK | t(9;12)(q22;p13) | MDS | ETV6/SYK | Abnormal activity of SYK leads to over- activation of JAK–STAT;  PI3K and MAPK pathways | Three cases | (34) |
| MDS1/EVI1 | t(3;12)(q26;p13) | MDS; CML; AML-M4 | ETV6/MDS1/EVI1 or ETV6/EVI1? | Overexpression of neighboring gene? | Five cases | (35-37) |
| MDS2 | t(1;12)(p36.1;p13) | MDS | ETV6/MDS2? | Overexpression of neighboring gene? | One case | (38) |
| FLT3 | t(12;13)(p13;q12) | MLN-Eo; MPN; MDS | ETV6/FLT3 | FLT3 activation | 18 cases | (39, 40) |
| LINC02260 | t(4;12)(q12;p13) | MPN-U | None | Unknown | One case | (41) |
| Abbreviations: ALL acute lymphoblastic leukemia, Pre-B ALL pre-B cell acute lymphoblastic leukemia, T-ALL T cell acute lymphoblastic leukemia, AML acute myeloid leukemia, MPAL mixed-phenotype acute leukemia, AEL acute erythroleukemia, MPN myeloproliferative neoplasm, MPN-U MPN-unclassifiable, MDS myelodysplastic syndrome, CML chronic myeloid leukemia, CMML chronic myelomonocytic leukemia,MLN-Eo myeloid/lymphoid neoplasm with eosinophilia. | | | | | | |

**References**

1. Østergaard A, Fiocco M, de Groot-Kruseman H, Moorman AV, Vora A, Zimmermann M, et al. ETV6::RUNX1 Acute Lymphoblastic Leukemia: how much therapy is needed for cure? Leukemia. 2024;38(7):1477-87.

2. Smeenk L, Fischer M, Jurado S, Jaritz M, Azaryan A, Werner B, et al. Molecular role of the PAX5-ETV6 oncoprotein in promoting B-cell acute lymphoblastic leukemia. Embo j. 2017;36(6):718-35.

3. Fouad FM, Eid JI. PAX5 fusion genes in acute lymphoblastic leukemia: A literature review. Medicine (Baltimore). 2023;102(20):e33836.

4. Strehl S, König M, Dworzak MN, Kalwak K, Haas OA. PAX5/ETV6 fusion defines cytogenetic entity dic(9;12)(p13;p13). Leukemia. 2003;17(6):1121-3.

5. Suto Y, Sato Y, Smith SD, Rowley JD, Bohlander SK. A t(6;12)(q23;p13) results in the fusion of ETV6 to a novel gene, STL, in a B-cell ALL cell line. Genes Chromosomes Cancer. 1997;18(4):254-68.

6. Qiao Y, Ogawa S, Hangaishi A, Yuji K, Izutsu K, Kunisato A, et al. Identification of a novel fusion gene, TTL, fused to ETV6 in acute lymphoblastic leukemia with t(12;13)(p13;q14), and its implication in leukemogenesis. Leukemia. 2003;17(6):1112-20.

7. Yagasaki F, Wakao D, Yokoyama Y, Uchida Y, Murohashi I, Kayano H, et al. Fusion of ETV6 to fibroblast growth factor receptor 3 in peripheral T-cell lymphoma with a t(4;12)(p16;p13) chromosomal translocation. Cancer Res. 2001;61(23):8371-4.

8. Maeda T, Yagasaki F, Ishikawa M, Takahashi N, Bessho M. Transforming property of TEL-FGFR3 mediated through PI3-K in a T-cell lymphoma that subsequently progressed to AML. Blood. 2005;105(5):2115-23.

9. Cook JR, Rogers HJ, Chandra PK, Prescott JL, Mukherjee S. Myeloid neoplasm with eosinophilia and ETV6-JAK2 fusion. Leuk Lymphoma. 2020;61(1):213-6.

10. Li HD, Chen SS, Ding J, Zhang CL, Qiu HY, Xia XX, et al. Exploration of ETV6::ABL1-positive AML with concurrent NPM1 and FLT3-ITD mutations. Ann Hematol. 2024;103(10):4295-304.

11. Griesinger F, Janke A, Podleschny M, Bohlander SK. Identification of an ETV6-ABL2 fusion transcript in combination with an ETV6 point mutation in a T-cell acute lymphoblastic leukaemia cell line. Br J Haematol. 2002;119(2):454-8.

12. Zhou MH, Gao L, Jing Y, Xu YY, Ding Y, Wang N, et al. Detection of ETV6 gene rearrangements in adult acute lymphoblastic leukemia. Ann Hematol. 2012;91(8):1235-43.

13. Iijima Y, Ito T, Oikawa T, Eguchi M, Eguchi-Ishimae M, Kamada N, et al. A new ETV6/TEL partner gene, ARG (ABL-related gene or ABL2), identified in an AML-M3 cell line with a t(1;12)(q25;p13) translocation. Blood. 2000;95(6):2126-31.

14. Silvertown JD, Lisle C, Semenuk L, Knapp C, Jaynes J, Berg D, et al. Prevalence of NTRK Fusions in Canadian Solid Tumour Cancer Patients. Mol Diagn Ther. 2023;27(1):87-103.

15. Roberts KG, Janke LJ, Zhao Y, Seth A, Ma J, Finkelstein D, et al. ETV6-NTRK3 induces aggressive acute lymphoblastic leukemia highly sensitive to selective TRK inhibition. Blood. 2018;132(8):861-5.

16. Otsubo K, Kanegane H, Eguchi M, Eguchi-Ishimae M, Tamura K, Nomura K, et al. ETV6-ARNT fusion in a patient with childhood T lymphoblastic leukemia. Cancer Genet Cytogenet. 2010;202(1):22-6.

17. Salomon-Nguyen F, Della-Valle V, Mauchauffe M, Busson-Le Coniat M, Ghysdael J, Berger R, Bernard OA. The t(1;12)(q21;p13) translocation of human acute myeloblastic leukemia results in a TEL-ARNT fusion. Proc Natl Acad Sci U S A. 2000;97(12):6757-62.

18. Fang F, Jia R, Liu C, Zhao H, Sun W. Genetic analysis and clinical significance of a rare t(1;12)(q21;p13) in a patient with high-risk myelodysplastic syndrome. Mol Genet Genomic Med. 2022;10(4):e1893.

19. Libbrecht C, Xie HM, Kingsley MC, Haladyna JN, Riedel SS, Alikarami F, et al. Menin is necessary for long term maintenance of meningioma-1 driven leukemia. Leukemia. 2021;35(5):1405-17.

20. Shao H, Cen J, Chen S, Qiu H, Pan J. Myeloid neoplasms with t(12;22)(p13;q12)/MN1-EVT6: a systematic review of 12 cases. Ann Hematol. 2018;97(3):417-24.

21. Cools J, Bilhou-Nabera C, Wlodarska I, Cabrol C, Talmant P, Bernard P, et al. Fusion of a novel gene, BTL, to ETV6 in acute myeloid leukemias with a t(4;12)(q11-q12;p13). Blood. 1999;94(5):1820-4.

22. Ma L, Yue Y, Zhang X, Wu Z, Wang W, Wang W. Acute myeloid leukemia with ETV6::CHIC2 fusion gene: 'Pitfalls' in diagnosis. Hematology. 2024;29(1):2381170.

23. Chase A, Reiter A, Burci L, Cazzaniga G, Biondi A, Pickard J, et al. Fusion of ETV6 to the caudal-related homeobox gene CDX2 in acute myeloid leukemia with the t(12;13)(p13;q12). Blood. 1999;93(3):1025-31.

24. Beverloo HB, Panagopoulos I, Isaksson M, van Wering E, van Drunen E, de Klein A, et al. Fusion of the homeobox gene HLXB9 and the ETV6 gene in infant acute myeloid leukemias with the t(7;12)(q36;p13). Cancer Res. 2001;61(14):5374-7.

25. Park J, Kim M, Lim J, Kim Y, Han K, Lee J, et al. Three-way complex translocations in infant acute myeloid leukemia with t(7;12)(q36;p13): the incidence and correlation of a HLXB9 overexpression. Cancer Genet Cytogenet. 2009;191(2):102-5.

26. Xu W, Tian F, Tai X, Song G, Liu Y, Fan L, et al. ETV6::ACSL6 translocation-driven super-enhancer activation leads to eosinophilia in acute lymphoblastic leukemia through IL-3 overexpression. Haematologica. 2024;109(8):2445-58.

27. Wu X, Cai H, Qiu Y, Li J, Zhou DB, Cao XX. ETV6-ACSL6 fusion gene in myeloid neoplasms: clinical spectrum, current practice, and outcomes. Orphanet J Rare Dis. 2020;15(1):192.

28. Zhang T, Wang Q, Xu Y, Wang M, Ma Z, Pan J, Chen S. ETV6::ACSL6 fusion gene in myeloid malignancies with eosinophilia: a report of two cases with t(5;12) or normal karyotype. Leuk Lymphoma. 2023;64(1):225-9.

29. Murga Penas EM, Cools J, Algenstaedt P, Hinz K, Seeger D, Schafhausen P, et al. A novel cryptic translocation t(12;17)(p13;p12-p13) in a secondary acute myeloid leukemia results in a fusion of the ETV6 gene and the antisense strand of the PER1 gene. Genes Chromosomes Cancer. 2003;37(1):79-83.

30. Mueller SB, Dal Cin P, Le LP, Dias-Santagata D, Lennerz JK, Iafrate AJ, et al. t(4;12)(q12;p13) ETV6-rearranged AML without eosinophilia does not involve PDGFRA: relevance for imatinib insensitivity. Blood Adv. 2022;6(3):818-27.

31. Gou Y, Tang Y, Liu S, Cheng S, Deng X, Wen Q, et al. Myeloid/Lymphoid Neoplasms with ETV6::PDGFRB Fusion Gene: A Rare Case of Poor Response to Imatinib and Possible Transformation Mechanisms from Myeloid Neoplasms of Bone Marrow to T-Cell Lymphoblastic Lymphoma Invasion in Lymph Nodes. J Inflamm Res. 2023;16:5163-70.

32. Pozdnyakova O, Orazi A, Kelemen K, King R, Reichard KK, Craig FE, et al. Myeloid/Lymphoid Neoplasms Associated With Eosinophilia and Rearrangements of PDGFRA, PDGFRB, or FGFR1 or With PCM1-JAK2. Am J Clin Pathol. 2021;155(2):160-78.

33. Golub TR, Barker GF, Lovett M, Gilliland DG. Fusion of PDGF receptor beta to a novel ets-like gene, tel, in chronic myelomonocytic leukemia with t(5;12) chromosomal translocation. Cell. 1994;77(2):307-16.

34. Manuelyan K, Momcheva I, Angelova S, Nikolov K, Shivarov V. Recurrent ETV6::SYK rearrangement in myeloid malignancies confers partial susceptibility to MEK inhibition. Br J Haematol. 2024;205(1):382-6.

35. Yamamoto K, Yakushijin K, Ichikawa H, Okamura A, Nagao S, Kakiuchi S, et al. Coexpression of ETV6/MDS1/EVI1 and ETV6/EVI1 fusion transcripts in acute myeloid leukemia with t(3;12)(q26.2;p13) and thrombocytosis. Leuk Lymphoma. 2019;60(5):1294-8.

36. Achkar WA, Aljapawe A, Liehr T, Wafa A. De novo acute myeloid leukemia subtype-M4 with initial trisomy 8 and later acquired t(3;12)(q26;p12) leading to ETV6/MDS1/EVI1 fusion transcript expression: A case report. Oncol Lett. 2014;7(3):787-90.

37. Peeters P, Wlodarska I, Baens M, Criel A, Selleslag D, Hagemeijer A, et al. Fusion of ETV6 to MDS1/EVI1 as a result of t(3;12)(q26;p13) in myeloproliferative disorders. Cancer Res. 1997;57(4):564-9.

38. Odero MD, Vizmanos JL, Román JP, Lahortiga I, Panizo C, Calasanz MJ, et al. A novel gene, MDS2, is fused to ETV6/TEL in a t(1;12)(p36.1;p13) in a patient with myelodysplastic syndrome. Genes Chromosomes Cancer. 2002;35(1):11-9.

39. Schoelinck J, Gervasoni J, Guillermin Y, Beillard E, Pissaloux D, Chassagne-Clement C. T cell phenotype and lack of eosinophilia are not uncommon in extramedullary myeloid/lymphoid neoplasms with ETV6::FLT3 fusion: a case report and review of the literature. Virchows Arch. 2024;484(5):853-7.

40. Spitzer B, Dela Cruz FS, Ibanez Sanchez GD, Zhang Y, Xiao W, Benayed R, et al. ETV6-FLT3-positive myeloid/lymphoid neoplasm with eosinophilia presenting in an infant: an entity distinct from JMML. Blood Adv. 2021;5(7):1899-902.

41. Zhang L, Wang M, Wang Z, Zeng Z, Wen L, Xu Y, et al. Identification of a novel ETV6 truncated fusion gene in myeloproliferative neoplasm, unclassifiable with t(4;12)(q12;p13). Ann Hematol. 2020;99(10):2445-7.
